# Is the scalar property of interval timing preserved after hippocampus lesions?

**DOI:** 10.1016/j.jtbi.2021.110605

**Published:** 2021-01-26

**Authors:** Tristan Aft, Sorinel A. Oprisan, Catalin V. Buhusi

**Affiliations:** a Department of Physics and Astronomy, College of Charleston, United States; b Department of Psychology, Utah State University, United States

**Keywords:** Hippocampus lesions, Computer model, Time cells, Striatal beat frequency model, Morris-Lecar neural model

## Abstract

Time perception is fundamental for decision-making, adaptation, and survival. In the peak-interval (PI) paradigm, one of the critical features of time perception is its scale invariance, i.e., the error in time estimation increases linearly with the to-be-timed interval. Brain lesions can profoundly alter time perception, but do they also change its scalar property? In particular, hippocampus (HPC) lesions affect the memory of the reinforced durations. Experiments found that ventral hippocampus (vHPC) lesions shift the perceived durations to longer values while dorsal hippocampus (dHPC) lesions produce opposite effects. Here we used our implementation of the Striatal Beat Frequency (SBFML) model with biophysically realistic Morris-Lecar (ML) model neurons and a topological map of HPC memory to predict analytically and verify numerically the effect of HPC lesions on scalar property. We found that scalar property still holds after both vHPC and dHPC lesions in our SBFML-HPC network simulation. Our numerical results show that PI durations are shifted in the correct direction and match the experimental results. In our simulations, the relative peak shift of the behavioral response curve is controlled by two factors: (1) the lesion size, and (2) the cellular-level memory variance of the temporal durations stored in the HPC. The coefficient of variance (CV) of the behavioral response curve remained constant over the tested durations of PI procedure, which suggests that scalar property is not affected by HPC lesions.

## 1. Introduction

Interval timing is defined as the perception and use of durations in the seconds to minutes range (Buhusi and Meck, 2010; Church, 2003; Church and Broadbent, 1990, 1991; Gallistel, 1990; Gallistel and Gibbon, 2000; Gibbon et al., 1988; Gibbon and Allan, 1984; MacDonald et al., 2014; Meck, 1996; Church, 1984). This process is critical for many essential behaviors, such as decision-making, rate calculation, and planning of action (Gallistel, 1990).

**Fig. 1.**
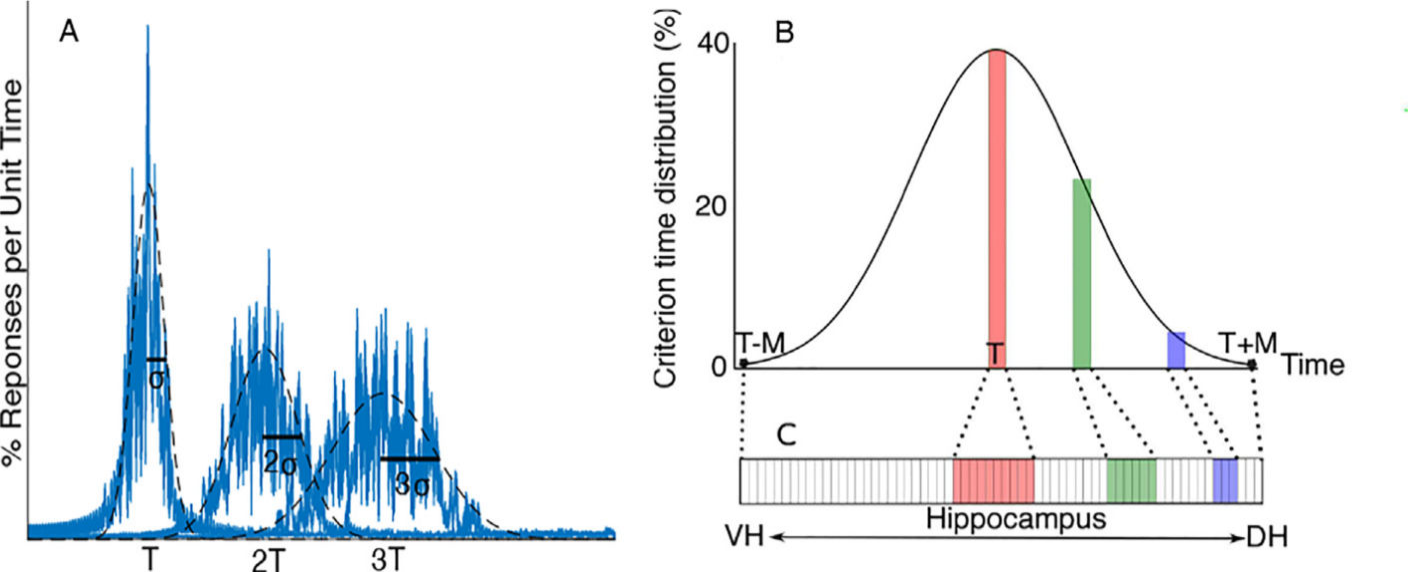
(A) Numerical simulations of peak-interval (PI) procedures using our Striatal Beat Frequency (SBFML) with biophysically-realistic Morris-Lecar (ML) model neurons (Oprisan and Buhusi, 2011; Oprisan et al., 2014; Oprisan and Buhusi, 2013) indicate that SBFML mimics the scalar property of interval timing. In our example, the criterion time is *T* = 10 s. The width *σ*_*behav*_ of the behavioral response curve scales linearly with the criterion time. Graphical representation of the two basic assumptions of this study: (B) First, at cellular-level, the long-term memory for durations is Gaussian with the peak around the criterion time *T* and a standard deviation *σ*_*mem*_. The area under each shaded rectangle represents the number of memory cells that store temporal duration values in the respective ranges. (C) The memorized durations are ordered from low to high and mapped onto HPC cells along the ventral (short durations) – dorsal (long durations) hippocampus. Each rectangle along the vHPC-dHPC line represents a memory cell. The number of allocated memory cells is proportional to the corresponding areas under the Gaussian curve shown in (B).

One of the main ways interval timing is experimentally tested is with the peak-interval (PI) procedure (Rakitin et al., 1998; Buhusi and Meck, 2006; Buhusi and Meck, 2010). In the PI procedure, a test subject is trained to associate a conditioning stimulus with a reward, but only after a certain time delay. For example, a rodent could be trained by rewarding it with food if it presses a lever *T* second after a conditioning stimulus (CS), which could be a light or a sound, is turned off. This time delay *T* is referred to as the criterion time. After training, the subjects are tested by having the CS presented with no reward, while their responses are recorded over time (Buhusi and Meck, 2005; Buhusi and Aziz, 2009). With no other manipulations present, the average behavioral response in the PI procedure usually yields a Gauss-like curve that peaks at the criterion time *T* (see Fig. 1A). When testing different criteria, the standard deviation *σ*_*behav*_ of the behavioral response curve linearly increases with the criterion time *T* (see Fig. 1A). As a result, when multiple behavioral response curves, such as those shown in Fig. 1A, are normalized along the temporal axis by their corresponding criterion time, they overlap (not shown). The linear relationship between the behavioral response curve width and the mean (peak) duration is known as *the scalar property or time*-*scale invariance* (Buhusi and Meck, 2010), as shown in Fig. 1A. The scalar property is present in many species (Gallistel, 1990; Buhusi and Meck, 2005), from invertebrates (Boisvert and Sherry, 2006), fish and birds, to mammals such as mice (Buhusi and Aziz, 2009), rats (Matell et al., 2004), and humans (Buhusi and Cordes, 2011; Rakitin et al., 1998; Hinton et al., 1996). It also holds under behavioral (Aitkin et al., 1970), lesion (Meck et al., 1987), pharmacological (Buhusi and Meck, 2002, 2010; Oprisan and Buhusi, 2011), and neurophysiological manipulations (Buhusi, 2000; Oprisan et al., 2014). Due to its consistency and ubiquity, time-scale invariance is seen as a fundamental property of interval timing.

### 1.1. Hippocampal lesions and interval timing

Recent work has investigated the role of HPC in episodic timing (MacDonald et al., 2014; Heys and Dombeck, 2018; Tsao et al., 2018; O’Neill et al., 2017). In regard to episodic timing, the HPC is involved with the encoding of temporal information in the Lateral Entorhinal Cortex (LEC) (MacDonald et al., 2014; Heys and Dombeck, 2018; Tsao et al., 2018; Buhusi, 2020). Since HPC is the site of spatial–temporal interaction, it also provides a basis for generation, maintenance and retrieval of episodic memories (MacDonald et al., 2014; Dickerson and Eichenbaum, 2010; MacDonald, 2014; Meck and Yin, 2014). While episodic timing is concerned with the sequence of events in time, interval timing is concerned with metric time, which is the estimation of temporal durations. In regard to interval timing, the HPC is involved with storing memory for durations in long-term memory substrates, since HPC lesions shift the peak of the behavioral response in PI procedures (MacDonald et al., 2014; Meck, 1996; Buhusi and Meck, 2005; Meck and Yin, 2014; Howard and Eichenbaum, 2013; Meck et al., 1984, 2013; Tam et al., 2015; Yin and Troger, 2011).

Lesion studies suggest that duration encoding may be spatially localized in the HPC. Rats with dorsal hippocampus (dHPC) lesions respond earlier than the trained duration (Meck et al., 1984, 2013; Balci et al., 2009; Merchant et al., 2013; Tam and Bonardi, 2012a,b; Tam et al., 2013, 2015). In contrast, ventral hippocampus (vHPC) lesions cause rats to respond later (Balci et al., 2009). The encoded durations could be mapped by the HPC for long-term storage to the LEC or in other log-term memory structures.

The present study integrates a topological map of the HPC (Oprisan et al., 2018a,b) with our implementation the SBFML interval timing model (Oprisan and Buhusi, 2011, 2013, 2014). In this topological map of the HPC, spatially localized time cells in the HPC (Kraus et al., 2013; MacDonald et al., 2011) are tied to stored values in the memory register shown in Fig. 1B. For a more detailed description of temporal cell mapping and its experimental justification see Oprisan et al. (2018a,b).

### 1.2. Topological maps across the brain

Many areas of the brain have been shown to have a topological organization. For example, grid cells in layer II of the entorhinal cortex, which provides information about an animal’s positioning, have differing subthreshold oscillation frequencies based on their location along the dorsal–ventral axis (Giocomo et al., 2007). The dorsocaudal medial entorhinal cortex has a directionally oriented, and topographically organized neural map of the spatial environment (Hafting et al., 2005). In the CA1 area of the HPC, gamma oscillations split into distinct fast and slow frequency components that differentially couple CA1 to inputs from the medial entorhinal cortex (Colgin et al., 2009). Jung et al. (1994) advanced the hypothesis of a hierarchical organization of spatial representation in the HPC. They identified experimentally a significantly smaller number of “place fields” in the vHPC than the dHPC, and the average spatial selectivity was of substantially lower resolution than in dHPC. Among the possible functional interpretations, they suggested (1) “a computational advantage of representing space at different scales”, which could hint at a free-scale fractal representation of spatial dimension of the environment, and (2) “a preeminence of essentially nonspatial information processing in the ventral hippocampus”, which we further explored in this study of temporal memories of events stored along ventral-dorsal HPC. A recent experimental study of theta rhythm in the HPC found that the theta phase-shifted monotonically with distance along the dorsoventral axis of the HPC (Patel et al., 2012). The authors concluded that “theta oscillations can temporally combine or segregate neocortical representations” along the dorsoventral axis of the HPC. An immunohistochemical investigation of protein expression of dopamine D2-like receptors (D2R) along the dorsoventral hippocampal axis identified significantly higher protein expression levels in the vHPV than the dHPC (Dubovyk and Manahan-Vaughan, 2019). The authors suggested that the gradient of D2R expression levels along the dorsoventral axis of the HPC “may support behavioral information processing by the ventral hippocampus.” It may also be possible that the gradient of D2R expression modulates the time cells of the HPC and leads to the observed gradient in the peak firing time of the spatially localized time cells (Kraus et al., 2013; MacDonald et al., 2011).

One could argue that topological maps in the brain optimize wiring lengths. Indeed, it is both more metabolically and structurally efficient to represent nearby feature space points by mapping them to neurons near that are close to each other rather than using long-range neural projections across different areas of the brain (Waissi and Rossin, 1996). Spatial localization also produces small local gradients of neural activity, which promote functional redundancy and reduce the spectral leakage between adjacent neurons.

In regard to timing durations, in the present study we assume a topological map of the HPC in that neurons that are spatially close together represent durations that are close together (see the dotted lines that project Gaussian distributed durations from Fig. 1B to spatially close memory locations). Based on our previous studies (Oprisan et al., 2018a,b), the Gaussian distribution of memorized times is mapped spatially along the ventral (short durations) – dorsal (longer durations) line of the HPC as shown in Fig. 1B.

## 2. Modeling the effect of hippocampal lesions on interval timing

To understand the effects of HPC lesions on the peak location and scalar property in PI procedures, we introduce first a mathematical framework that accurately incorporates the results from experimental studies. Our numerical simulations are grounded in the SBFML model (Buhusi and Oprisan, 2013; Oprisan and Buhusi, 2011, 2013; Buhusi et al., 2016; Oprisan and Buhusi, xxxx). The SBFML model origin can be traced back to Meck and co-workers (Buhusi and Meck, 2005; Matell and Meck, 2004) who showed that time could be coded by the coincidental activation of neurons, which produces firing beats with periods spanning a much wider range of durations than single neurons (Miall, 1989). Repeatable patterns of neural oscillations in the alpha band (8 Hz to 12 Hz) of the electroencephalogram (Anliker, 1963 and reseting of oscillatory activity in neocortex during timing tasks (Rizzuto et al., 2003), are among the supporting evidences of the SBFML neural oscillators model ability to form representations of temporal durations (Matell et al., 2005). While in the SBFML cortical oscillators of various frequency are most likely located in the prefrontal cortex (PFC) (Buhusi and Oprisan, 2013; Oprisan and Buhusi, 2011, 2013; Buhusi et al., 2016; Oprisan and Buhusi, xxxx), other cortical regions could also be involved (Kononowicz and van Wassenhove, 2016; Suzuki and Tanaka, 2019). The states of cortical oscillators at the reinforcement (criterion) time could be stored in the HPC (Lisman and Grace, 2005) and the striatum, which we mimic in the SBFML-HPC model by a memory register similar to the one shown in Fig. 1B. In the SBFML implementation, the comparison between a stored representation of an event, e.g. the set of the states of cortical oscillators at the reinforcement (criterion) time *T*, and the current state of the same cortical oscillators during the ongoing test trial is performed by the striatal spiny neurons (Hinton et al., 1996; Wilson, 1995; Stern et al., 1998; Chiba et al., 2008; Doig et al., 2010; Harrington and Jahanshahi, 2016). The output from the spiny neurons mimics the Gaussian shape of behavioral response curve shown in Fig. 1A.

**Fig. 2.**
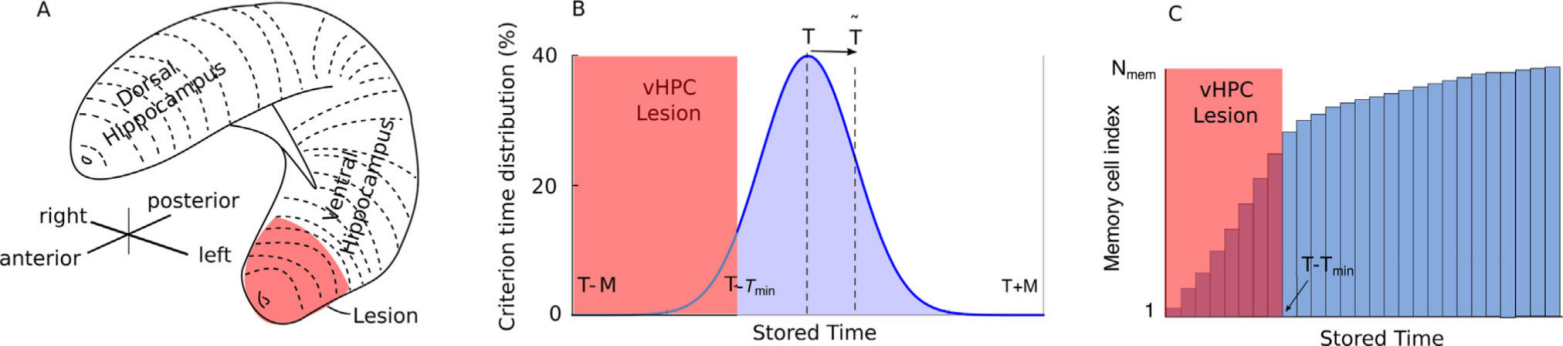
Modeling Hippocampal Lesions. (A) A sketch of the three-dimensional organization of a rodent’s HPC. The symmetric Gaussian distribution of memorized durations maps along the HPC with shorter durations stored in the vHPC and longer durations stored in the dHPC. (B) A ventral hippocampal lesion (shaded area) biases the memory content towards longer durations. Before lesion, the criterion time was normally distributed with the probability distribution function $pdf(\mu =T,\sigma )=\frac{1}{\sqrt{2\pi \sigma }}e^{\frac{{\left(x-\mu \right)}^{2}}{2\sigma^{2}}}$ over the symmetric range (*T* – *M*, *T* + *M*) and peaked at *T* (blue curve). After the vHPC lesion, the memory cells between (*T* – *M*, *T* − *T*_*min*_) were removed, and the new distribution peaks at $\tilde{T}>T$. (C) Due to the memory’s topological organization, lesions reduce the actual memory size and produce a non-symmetric memory of learned criterion time. The cumulative distribution (panel C) gives the number of allocated memory cells to storing a specific range of durations and was computed by integrating the Gaussian distribution in panel (B).

In our model, HPC lesions are represented by the dark shaded area, as shown in Fig. 2A, where vHPC was lesioned. Since the HPC memory is finite, there is a maximum, limited range *M* of HPC stored values around the criterion time *T*. The lesion removed the short durations (shaded area) from the initially symmetric Gaussian distribution of memorized durations that covered the range (*T* – *M*, *T* + *M*) (see Fig. 2B). Since there are a finite number of memory cells in both our simulations and in the subject’s HPC, the theoretically continuous Gaussian distribution of durations is implemented as a discrete set of values stored in HPC memory cells (see Fig. 2C). The shaded area under the Gaussian curve in Fig. 1B give the number of memory cells that store the respective values. The number of HPC memory cells allocated (see Fig. 1B) to holding a specific value of the reinforced duration is proportional to the likelihood of observing the respective duration. The selective deletion of some memory cells due to lesions results in a non-symmetric temporal memory over the temporal durations *T* − *T*_*min*_, *T* + *M* (see Fig. 2). Although we only refer to vHPC lesions in the following, our results can be immediately transferred to dHPC lesions due to the symmetry of temporal mapping (see Fig. 2B).

## 3. Results

We first made a series of analytical predictions regarding the shift in peak time and the coefficient of variance *CV* of the behavioral response curve in the PI experiments after HPC lesions (see Section 3.1). The predictions were then checked against numerical simulations done with the SBFML model (see Section 3.2) and against the existing literature on HPC lesions (see Section 3.3). For a given vHPC lesion, we considered that the fraction of lesioned HPC only runs from 0% (no lesion) to 50% when the lesion covers entirely the vHPC and extends up to the midline between the vHPC and dHPC (see Figs. 1B and 2). This notation agrees with the experiments on HPC lesions that report the percentage of vHPC or dHPC lesions with respect to the total HPC area (see Section 3.3).

### 3.1. Analytical predictions

To predict theoretically the shift in the mean (peak) of the behavioral response curve due to HPC lesions, we first estimate the range *M* of the durations stored in the memory register of Fig. 1C for a Gaussian distribution *N*(*T*,*σ*_*mem*_) with a mean criterion time *T* and a standard deviation *σ*_*mem*_ (see Fig. 1B). For any given criterion time *T*, the range *M* measures the spread of the Gaussian distribution of criteria and is determined by the standard deviation of memorized criteria stored at the cellular-level *σ*_*mem*_. Second, since there are a limited number of memory cells, the lesions will produce an output based on a subset (*T* − *T*_*min*_, *T* + *M*) of the original range (see the blue shaded area under the Gaussian curve in Fig. 2B). A vHPC lesion of size *n*_*vHPC*_ determines the lower limit *T*_*min*_ of memorized durations (see Fig. 2B and C). Since the distribution of memorized durations in the lesioned memory is not symmetrical (see the blue curve in Fig. 2B), it introduces a skewness that shifts the mean (peak) value from *T* (before lesions) to $\tilde{T}$ (after lesions). Similarly, the standard deviation ${\tilde{\sigma }}_{mem\;}$ of the memorized durations after vHPC lesion is different form the original standard deviation *σ*_*mem*_.

To simplify the derivations, we consider that the criterion time follows a normal distribution *N*(0, 1) with zero mean, *μ* = 0, and unity standard deviation, *σ* = 1, given by the Gaussian probability distribution function $pdf(\mu ,\;\sigma )=\frac{1}{\sqrt{2\pi }\sigma }e^{\frac{{\left(x-\mu \right)}^{2}}{2\sigma^{2}}}$. For an arbitrary criterion time *T* and an arbitrary standard deviation *σ*_*mem*_, a change of variable $Z=\frac{x-T}{\sigma_{mem}}$ transforms *N*(0, 1) to the probability distribution *N*(*T*,*σ*_*mem*_). Such change of variables allows us to derive all our results assuming the memorized durations are Gaussian with zero mean and unit standard deviation *N*(0, 1) and then generalize them to real-world criteria *T* and standard deviations *σ*. It is also important to note that although working with *N*(0, 1) is mathematically convenient, half of the distribution has negative values, which is not physically realistic when we refer to durations. This is another reason why the change of variable is important since it shifts the entire Gaussian distribution to real-world, positive, values of durations.

To answer the first question regarding the range *M* of durations stored in a finite memory register of size *N*_*mem*_ for a criterion time *T*, we used the extreme order statistics approach (Chen and Tyler, 1999). If Φ(*x*) is the cumulative distribution function *cdf*_*x*_ shown in Fig. 2C, then the maximum value *M* stored in this finite-size Gaussian distributed memory register (see Fig. 1C) can be approximated by $M=\Phi^{-1}\left(0.5264^{\frac{1}{N_{mem}}}\right)$, where *N*_*mem*_ is the number of samples, i.e., the HPC memory size. The accuracy of the approximation is 0.5% (Chen and Tyler, 1999). Since Φ(*x*) has no analytic solution, we used a sigmoidal approximation (Wilson and Bednar, 2015) that covers a wide range (|*x*| < 8) with good accuracy (better than 10^−5^):

(1) $$\Phi (x)=\frac{1}{1+e^{\sqrt{\pi }\left(\beta_{1}x^{5}+\beta_{2}x^{3}+\beta_{3}x\right)}}.$$

It is important to emphasize a few points: (1) “x” in the *cdf*_*x*_ in Eq. (1) is actually a duration since we are concerned with temporal distributions, (2) given that the distribution of durations is centered at zero with unit standard deviation, *N*(0, 1), the range |*x*| < 8 is eight time the standard deviation *σ*_*mem*_ = 1, which is why |*x*| < 8 is considered a wide range for Eq. (1).

**Fig. 3.**
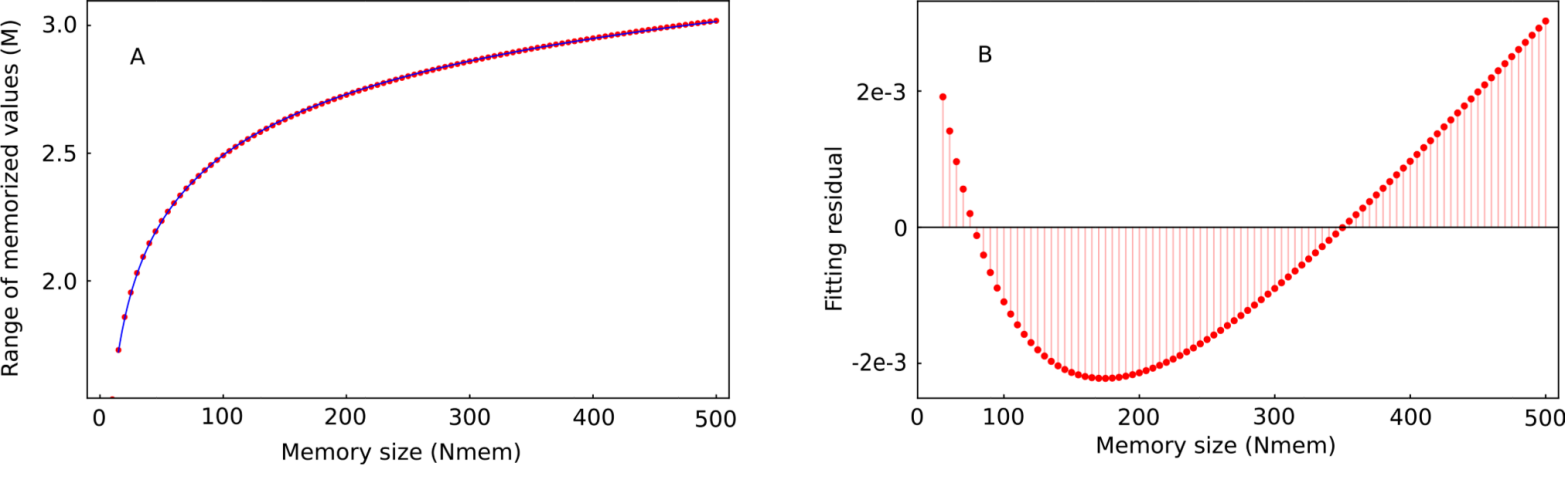
(A) Numerical solution of $\Phi (M)=0.5264^{1/N_{mem}}$ (solid circles) and the corresponding power-law fitting given by Eq. (2) (continuous line). (B) The fitting residual is below 0.1% of *M*.

After numerically solving the equation $\Phi (M)=0.5264^{1/N_{mem}}$, we found a simplified analytical expression for the range of memorized criteria (see Fig. 3A):

(2) $$M=\left((5.340\pm 0.025)+(-5.099\pm 0.019)N_{mem\;}^{-0.1260\pm 0.0011}\right)\sigma_{mem\;}.$$

The number of memory cells (*N*_*mem*_ < 500) for our data fitting model given by Eq. (2) is consistent with the numerical simulations (see Section 3.2). For data from *N*(0, 1), the goodness of fit for Eq. (1) is high with a fitting residual below 0.1% of *M* (see Fig. 3B). Besides providing a convenient formula for estimating the range *M* of stored values as a function of the memory size *N*_*mem*_, (2) also shows that the range *M* is proportional to the cellular-level standard deviation *σ*_*mem*_ of the Gaussian distribution of criteria.

To answer the second question regarding the lower limit of memorized durations *T*_*min*_ after a vHPC lesion, we used the cumulative distribution function Φ(*x*) again. The *cdf*_*x*_ has a few properties we use below: (1) the area under the curve is always unity (see Fig. 2C), (2) for a symmetric Gaussian distribution *N*(0, 1), the sum of the areas over the negative range, Φ(−*x*), plus the area over the positive range, Φ(*x*), gives unit area, i.e. Φ(−*x*) ) + Φ(*x*) = 1. By definition of the cumulative distribution function, the fraction of memory cells holding values *T* − *T*_*min*_ < *T* < *T* + *M* is:

(3) $$n_{vHPC}=\frac{N_{lesion\;}}{N_{mem\;}}=\frac{1}{\sqrt{2\pi }}\int_{T-T_{min}}^{T+M}pdf(0,\;1)dx\;\;=\Phi (T+M)-\Phi \left(T-T_{min}\right),$$

which gives the following estimate for Φ(*T*_*min*_) in the case of *N*(0, 1) distribution of durations:

(4) $$\Phi \left(T_{min}\right)=\frac{N_{lesion\;}}{N_{mem\;}}+\Phi (-M)=\frac{N_{lesion\;}}{N_{mem\;}}+1-\Phi (M).$$

The lower limit *T*_*min*_ of the stored durations after a HPC lesion is related to the pre-lesion range of memorized durations *M* and the lesion’s size *N*_*lesion*_/*N*_*mem*_. To solve Eq. 4, we used again the sigmoidal approximation given by Eq. 1 (Wilson and Bednar, 2015 and numerically determined *T*_*min*_ as functions of *n*_*vHPC*_ = *N*_*lesion*_/*N*_*mem*_ (not shown).

To answer the third question regarding the mean (peak) shift of the behavioral response curve to $\tilde{T}$ and the change in standard deviation to ${\tilde{\sigma }}_{behav\;}$ after a vHPC lesion, we used the spatial mapping of memorized durations along the HPC ventral-dorsal line (see Fig. 1B). For this purpose, from a normal distribution around a criterion time *T*, we take *N*_*mem*_ samples and map them in the long-term memory register (Fig. 1B) with the low values in the vHPC and the higher values in the dHPC as shown in Fig. 2. For example, for a vHPC lesion, we remove the lower portion of memory register (see Fig. 2). Similarly, for dHPC lesions, we remove higher values between (*T* – *M*, *T* + *T*_*max*_) from the long-term memory (not shown), and computed the upper limit *T*_*max*_.

**Fig. 4.**
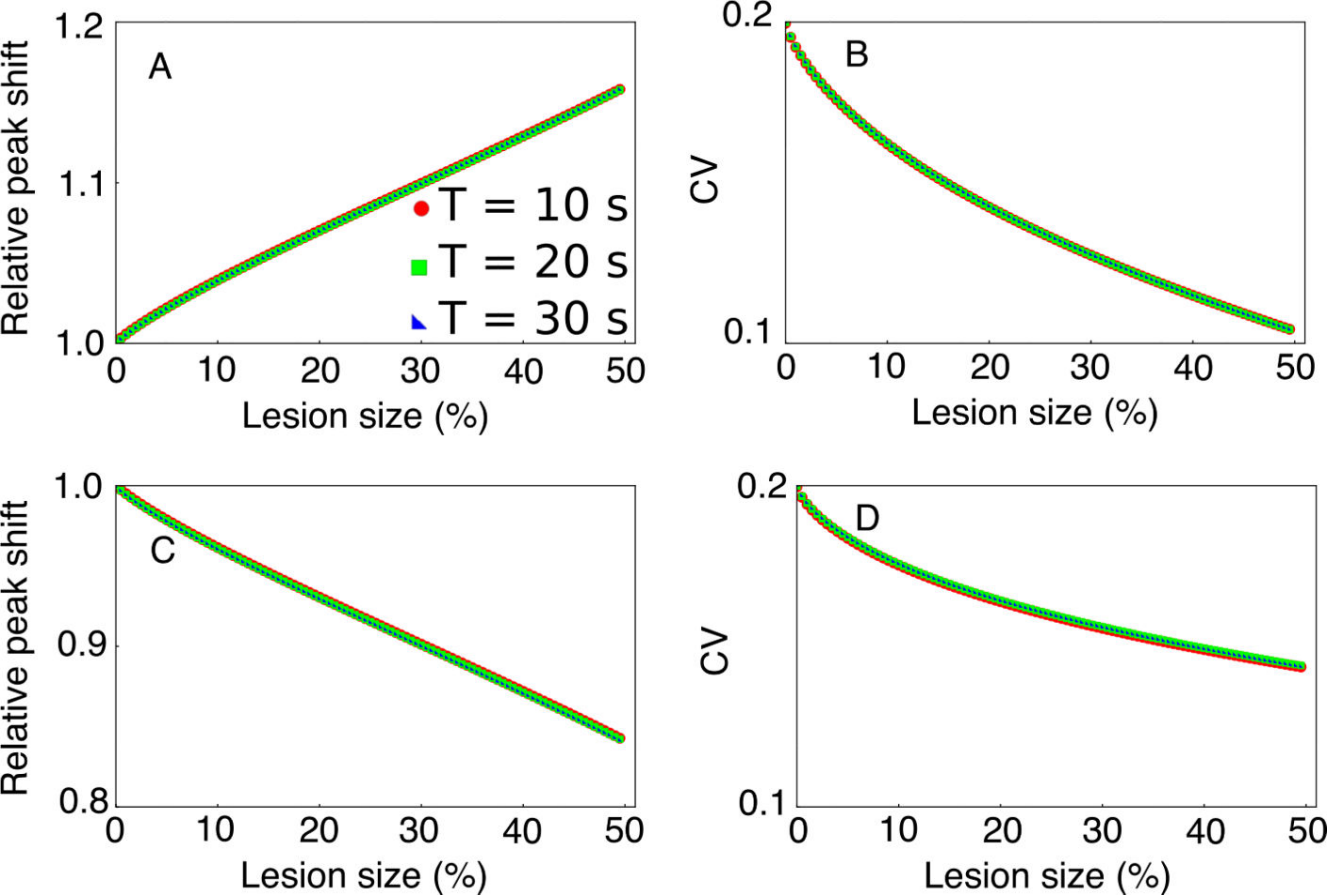
*Scalar property is maintained after HPC lesions*. (A) Numerically predicted values for the relative peak shift after vHPC lesion show an almost linear increase of peak shift with the lesion size afterward. (B) The corresponding *CV* has a nonlinearly decrease trend. (C) Dorsal HPC lesions show a steady leftward shift of PI peak towards lower durations as the lesion size increases. (D) The corresponding dHPC *CV* almost mirrors the vHPC predictions. The predictions of all three criteria overlap in all panels.

As one notices from Fig. 2B, a vHPC lesion shrinks the original distribution from the symmetrical range (*T* – *M*, *T* + *M*) to a narrower and asymmetric range (*T* − *T*_*min*_, *T* + *M*). We computed the new mean value of the distribution numerically after vHPC lesion, i.e.,

$$\bar{x}=\tilde{T}=\int_{T-T_{min}}^{T+M}xpdf\left(T,\sigma_{mem}\right)dx$$

and assign it to the new peak time $\tilde{T}$. The relative peak shift $\tilde{T}/T$ for 3 different criteria is shown in Fig. 4A. Similarly, the mean of the squares of the skewed distribution was numerically computed using

$$\bar{x^{2}}=\int_{T-T_{min}}^{T+M}x^{2}\;pdf\left(T,\sigma_{mem}\right)dx,$$

which gives the new standard deviation ${\tilde{\sigma }}_{behav}=\sqrt{\bar{x^{2}}-{\bar{x}}^{2}}$. The *CV*, which is ${\tilde{\sigma }}_{behav\;}/\tilde{T}$ is shown in Fig. 4B. Similar computations were done for dHPC lesions and the results are shown in Fig. 4C and D. As Fig. 4 shows, the peak shifts and *CV*s are independent of the criterion time. The fact that the *CV*s overlap for three different criteria supports our prediction that the scalar property is maintained after HPC lesions.

While the relative shift is roughly in the range of 15% of the criterion time both for vHPC (Fig. 4A) and dHPC (Fig. 4C) lesions, the *CV* for vHPC lesions (Fig. 4B) covers a wider range than for vHPC (Fig. 4D). This is because ${\tilde{T}}_{vHPC}$ is always larger than ${\tilde{T}}_{dHPC}$, which, for equal relative width of the behavioral response curve, leads to smaller *CV* values for vHPC lesions as shown in Fig. 4B.

To conclude our theoretical prediction section, the main predictions are that: (1) the magnitude of the peak shift is linearly increasing with the lesion size, (2) the *CV* has a strongly nonlinear dependence on the lesion size, and (3) both the mean (peak) shift and the CV are independent of the criterion time. *The fact that the CV for a given lesion size is independent of the criterion time means that the scalar property is valid after HPC lesions*.

### 3.2. Numerical verification of predictions

While our predictions in Section 3.1 do not depend on the particular implementation of the interval timing model, we used our previous implementation of the SBFML model to test our theoretical predictions (Oprisan et al., 2014; Oprisan and Buhusi, 2013). We used three different criteria *T* = 10 s, 20 s and 30 s, and three different pre-lesion memory sizes *N*_*mem*_ = 100, 200 and 300 cells for our simulated lesions. We used memory variance of 5%, 10%, 15%, 20%, 25% and 30% of the criterion time for each criterion time simulation. We used different memory variance values *σ*_*mem*_ (see Fig. 1B) because we predicted that variation in the encoded time at the cellular-level would be correlated with the standard deviation of the network-level measured peak width *σ*_*behav*_ (see Fig. 1A).

#### Peak shift and *CV* dependence on lesion size and criterion time.

**Fig. 5.**
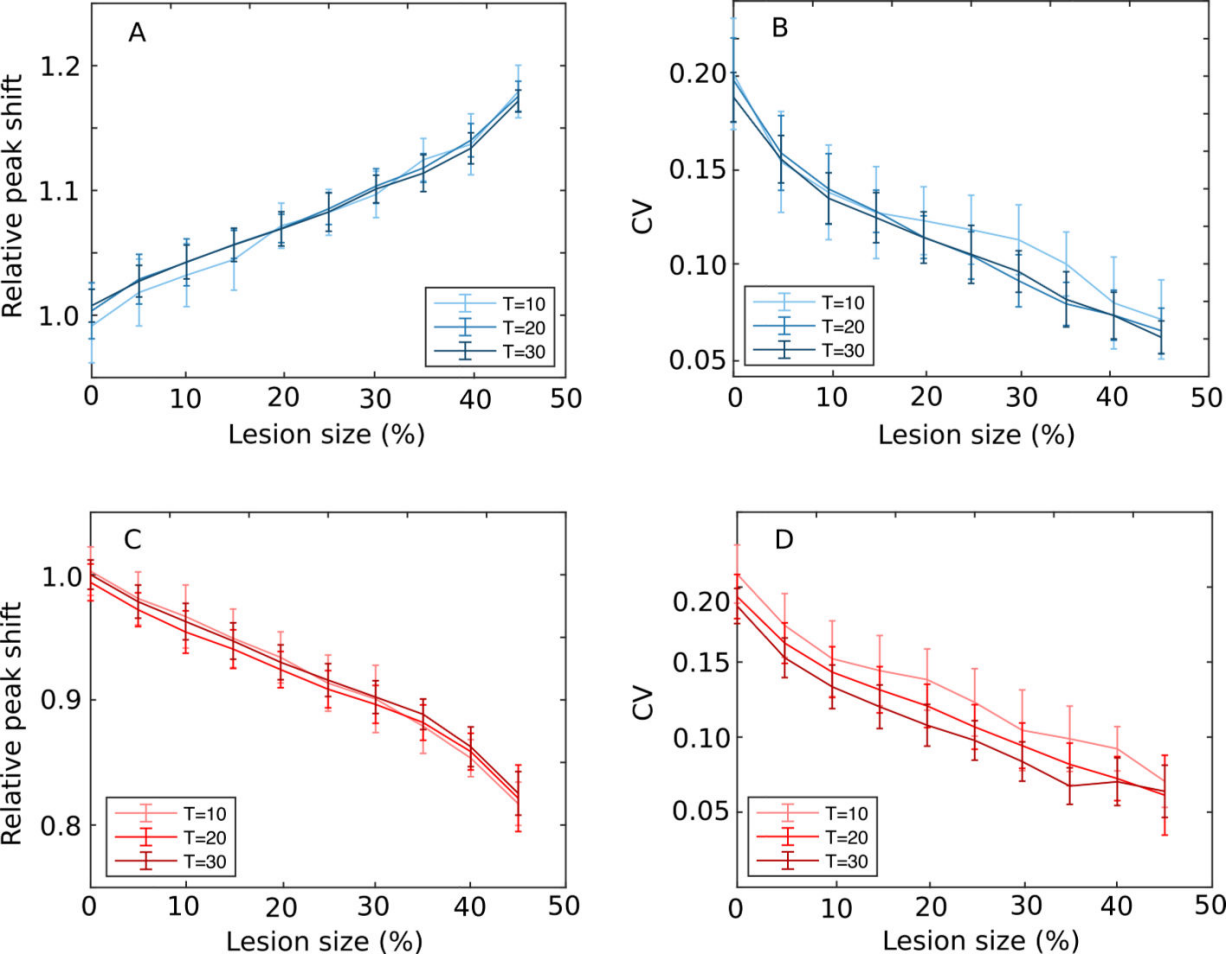
vHPC (top) and dHPC (bottom) simulated lesion using SBFML model for 3 criterion times, *T* = 10 s, 20 s, and 30 s, *N*_*mem*_ = 300 memory cells, *σ*_*mem*_ = 10% memory variance. Peak shifts (panels A and C) are normalized by criterion time. Peak response times were found to be significantly shifted for both vHPC (A) with about 20% and dHPC (C) lesions with about −20% for 10% memory variance. The overlap of the peak shifts for three criteria *T* = 10 s, 20 s and 30 s shows that the peak shift is always proportional to the lesion type’s criterion time. In both vHPC and dHPC lesions, the peak shift was also proportional to lesion size (panels A and C). The *CV* for both vHPC and dHPC lesions steadily decreases with increasing lesion size (panels B and D). These numerical results mirror the analytical predictions from Fig. 4.

We found that simulated lesions with the SBFML model significantly shift the peak of the behavioral response curve, as shown in Fig. 5A for vHPC and Fig. 5C for dHPC lesions. vHPC lesions shifted the peak response time later (positive relative shift in Fig. 5A), and dHPC lesions shifted the peak response time earlier (negative relative shift in Fig. 5C), consistent with experimental results (Tam and Bonardi, 2012a,b) (see also Section 3.3). As the lesion’s size increased, the peak shift’s magnitude increased linearly with the lesion size in both vHPC and dHPC lesions.

To test that timing remains scalar in the SBFML-HPC model after HPC lesions, we numerically evaluated the *CV* for each lesion size. For the interval timing to remain scalar, the *CV* must be constant over multiple criteria. We found that for both vHPC (see Fig. 5B) and dHPC (see Fig. 5D) lesions, the *CV* decreased linearly as the lesion size increases. At the same time, the *CV* curves for the three different criteria are identical, within the standard error. This shows that *CV* is indeed independent of the criterion time, which means the timing obeys scalar property for both vHPC and dHPC lesions.

Before comparing our numerical simulations against experimental data, we briefly review two of many experimental studies on HPC lesions and timing. Dorsal HPC lesions performed by Tam and Bonardi (2012a) measured a HPC damage of 34% of total HPC volume. Without repeating all the details of the different types of interval timing tasks, we summarize their results: (1) in Delay CS Peak Trials (see Fig. 5 of Tam and Bonardi, 2012a) they found a −25% peak shift from 20 s to 15 s in block 1 and a −37% peak shift from 19 s to 12 s in block 2; (2) in Trace CS off Peak Trials (see Fig. 5 of Tam and Bonardi, 2012a) they found a −11% peak shift from 27 s to 24 s in block 1 and a −22% peak shift from 32 s to 25 s in block 2, and (3) in nonreinforced peak trials (see Fig. 6 of Tam and Bonardi, 2012a) they found a −38% peak shift from 16 s to 10 s.

In another experimental study on mice with cytotoxic lesions of the hippocampus, Meck and Yin (2014) compared side by side the dHPC and vHPC lesions. They found that pre-training dHPC lesions underestimate target durations and preserve scalar property. The pre-training vHPC lesions produce rightward shift and violate scalar property. While peak shifts depend on the number of training sessions, for pre-training dHPC they found a −26% shits from 16.06 s to 11.89 s for a 15 s target time and a −23% shift from 46.78 s to 36.00 s for a 45 s target duration (see Table 2 in Meck and Yin, 2014). For vHPC lesions, the peak shift of −10% was found from 15.00 s to 13.46 s for a 15 s target duration and +5% shift from 44.10 s to 46.46 s for a 45 s target duration.

### 3.3. Comparison of numerical simulation against experimental data on hippocampus lesions

We also compared our numerical simulation results against experimental results from published papers on the effects of HPC lesions on interval timing. In a recent peak interval study on rats (Matell et al., 2014), the spread of the distribution was defined as the time delay between the point where the peak first reaches half of the maximal response and the point where it descends back down to half the maximal rate. With Gaussian distributions, this is the same as the full width at the half maximum (FWHM). The FWHM is related to the variance that we have been using in this study by *FWHM* = 2*ln*(2) · *σ* ≈ 2.3548*σ*. In the study (Matell et al., 2014), for a criterion time of *T* = 32.82 s, they found that *FWHM* = 23.65 s. Similarly, for *T* = 30.09 s, they find that *FWHM* = 29.24 s. As a result, the estimated standard deviation from behavioral experiments is *σ*_*behav*_ = (0.3–0.4)*T*.

In other studies (Tam and Bonardi, 2012a,b), the authors examined the effects of dHPC lesions on peak response procedures. They used an average dHPC lesion size of 38% and a criterion time *T* = 15 s. They found a shift to around 10 s, representing a 33.3% decrease. Their results for the FWHM were similar to Matell et al. (2014) study and gave a standard deviation of behavioral experiments *σ*_*behav*_ = (0.26–0.43)*T*.

We also notice that the results in two lesion papers (Tam and Bonardi, 2012a,b) are quite different. With the same size lesion of 38% and similar variance values, *σ*_*behav*_ = (0.26–0.43)*T* compared to *σ*_*behav*_ = (0.27–0.58)*T*, we get significantly different shifts. Regarding the first study by Tam and Bonardi (2012b), they measured a 33% peak shift. In the second study by Tam and Bonardi (2012a), they report a 7.75% to 8.25% peak shift. Our numerical results are off by a factor of 2.2–6.6 when compared against (Tam and Bonardi, 2012b). On the other hand, our numerical results agree with the peak shifts found in the latter study (Tam and Bonardi, 2012a) by the same authors. If we take the latter to be correct, we would need to have a lesion size larger than 41% to get the shift found in Tam and Bonardi (2012b). The discrepancy may be due to the fact that the results reported in Tam and Bonardi (2012b) were averaged over multiple lesion sizes.

## 4. Discussion

Experiments in rodents showed that dHPC lesions produced leftward (lower durations) shifts in peak interval procedures (Tam et al., 2013, 2015; Tam and Bonardi, 2012b), and vHPC lesions produced rightward shifts (Meck and Yin, 2014; Bannerman et al., 1999). We previously showed that the experimental observations support a temporal information model with a HPC topological map (Oprisan et al., 2018a,b). Here we carried out both analytical derivations and numerical simulations using the SBFML model augmented with a topological map of HPC memory (SBFML-HPC) to test the effect of HPC lesions on scalar property.

We theoretically predicted that *the shift in peak response time of the behavioral response curve would be proportional to lesion’s size* relative to the total memory size, and the direction was dependent on which side of the HPC the lesion was placed (see Section 3.1). We also predicted theoretically that *both dHPC and vHPC lesions should preserve scalar property*. These predictions were verified numerically (see Section 3.2) and found to agree with experimental results (see Section 3.3).

Others have experimentally examined the differing effects of vHPC and dHPC lesions, and found that the scalar property holds for dHPC lesions but not vHPC lesions (Meck and Yin, 2014). However, our simulations showed that both vHPC and dHPC lesions maintain the scalar property. One possible explanation of a non-scalar timing effect in experimental vHPC lesions reported in Meck and Yin (2014) could be that in our numerical simulations it is possible to selectively and precisely delete HPC cells, which is not necessarily feasible in neurotoxic studies. This could explain why vHPC lesions may appear to violate the scalar property while dHPC lesions still produce scalar timing in Meck and Yin (2014).

Our numerical simulations were compared against experimental results after HPC lesions and found strong agreement with results published in Matell et al. (2014). Two of the studies reported very different peak shifts after similar lesions (Tam and Bonardi, 2012a,b), and our simulations agreed with one of them (Tam and Bonardi, 2012b). The only observed disagreement is most likely due to the experimental methodology, i.e., the authors in Tam and Bonardi (2012a) may have averaged their results over different lesion size, which makes impossible a side-by-side comparison against numerical results.

Although this study builds on the HPC topological map idea (Oprisan et al., 2018a,b), it brings significant and novel findings. First, regarding the SBFML-HPC model implementation of simulated lesions in our previous studies, in the current study we chose a specific lesion size and varied the location around the HPC (Oprisan et al., 2018a,b). Improved spatial localization of HPC lesions in experimental studies allowed us to redesign our numerical approach to mirror biologically relevant findings. This new approach allowed us to compare our numerical results against the effect of experimental lesions with a specific size and an uncertain location.

Second, in our previous studies (Oprisan et al., 2018a,b), the predicted peak shifts for a given lesion size were not as large as those observed in experimental studies. We hypothesized that the difference was due to a larger variance (stronger biological noise at cellular-level) in the neurotoxic studies compared to our simulations. Since we previously fixed the memory variance at *σ*_*mem*_ = 10% and neurotoxic lesion studies found a peak width of *σ*_*behav*_ = 30%, our results could be scaled by a factor of 3. In this study, we found that the peak shift and the *CV* of the behavioral response curve remained constant over the numerically simulated durations of PI procedure, which suggests that scalar property is valid under HPC lesions.

Future work could expand on our hypothesis of a symmetric Gaussian distribution of memorized durations (see Fig. 2). Peak interval procedures in humans showed indeed a markedly symmetric Gaussian distribution of responses that justifies our hypothesis (Rakitin et al., 1998; Wearden and McShane, 1988). In contrast, rats and pigeons frequently produce asymmetrical, right-skewed functions described well by a Gaussian plus a ramp function (Roberts, 1981; Cheng and Westwood, 1993). The skewness observed in animals is presumably determined by responses not controlled by the timing task (Wearden and McShane, 1988), although we proposed that it could also be due to neural noise in firing frequency (Oprisan and Buhusi, 2014). In experiments where the skewness is significant and determined purely by the timing tasks, a generalized distribution that includes skewness could be used (Azzalini, 1985).
